# Are environmental risk factors for current wheeze in the International Study of Asthma and Allergies in Childhood (ISAAC) phase three due to reverse causation?

**DOI:** 10.1111/cea.13325

**Published:** 2019-01-23

**Authors:** Richard J. Silverwood, Charlotte E. Rutter, Edwin A. Mitchell, M. Innes Asher, Luis Garcia‐Marcos, David P. Strachan, Neil Pearce, the ISAAC Phase Three Study Group, N Aït‐Khaled, HR Anderson, R Beasley, B Björkstén, B Brunekreef, J Crane, P Ellwood, C Flohr, F Forastiere, S Foliaki, U Keil, CKW Lai, J Mallol, CF Robertson, S Montefort, J Odhiambo, J Shah, AW Stewart, D Strachan, E von Mutius, SK Weiland, G Weinmayr, G Wong, TO Clayton, CE Baena‐Cagnani, M Gómez, ME Howitt, J Weyler, R Pinto‐Vargas, AJ da Cunha, L de Freitas Souza, C Kuaban, A Ferguson, P Standring, P Aguilar, L Amarales, LA Benavides, Y‐Z Chen, O Kunii, Q Li Pan, NS Zhong, G Aristizábal, AM Cepeda, GA Ordoñez, C Bustos, M‐A Riikjärv, K Melaku, R Sa'aga‐Banuve, J Pekkanen, IE Hypolite, Z Novák, G Zsigmond, S Awasthi, S Bhave, NM Hanumante, KC Jain, MK Joshi, VA Khatav, SN Mantri, AV Pherwani, S Rego, M Sabir, S Salvi, G Setty, SK Sharma, V Singh, T Sukumaran, PS Suresh Babu, CB Kartasasmita, P Konthen, W Suprihati, MR Masjedi, A teriu, BN Koffi, H Odajima, JA al‐Momen, C Imanalieva, J Kudzyte, BS Quah, KH Teh, M Baeza‐Bacab, M Barragán‐Meijueiro, BE Del‐Río‐Navarro, R García‐Almaráz, SN González‐Díaz, FJ Linares‐Zapién, JV Merida‐Palacio, N Ramírez‐Chanona, S Romero‐Tapia, I Romieu, Z Bouayad, R MacKay, C Moyes, P Pattemore, BO Onadeko, G Cukier, P Chiarella, F Cua‐Lim, A Brêborowicz, G Lis, R Câmara, ML Chiera, JM Lopes dos Santos, C Nunes, J Rosado Pinto, E Vlaski, P Fuimaono, DY Goh, HJ Zar, HB Lee, A Blanco‐Quirós, RM Busquets, I Carvajal‐Urueña, G García‐Hernández, A ópez‐Silvarrey Varela, M Morales‐Suárez‐Varela, EG Pérez‐Yarza, OA Musa, O Al‐Rawas, S Mohammad, K Tabbah, JL Huang, CC Kao, M Trakultivakorn, P Vichyanond, T Iosefa, M Burr, D Holgado, MC Lapides, HH Windom, O Aldrey, D Solé, M Sears, S Barba, K Baratawidjaja, S Nishima, J de Bruyne, N Tuuau‐Potoi, CK Lai, BW Lee, A El Sony, R Anderson

**Affiliations:** ^1^ Department of Medical Statistics London School of Hygiene and Tropical Medicine London UK; ^2^ Department of Paediatrics: Child and Youth Health Faculty of Medical and Health Sciences University of Auckland Auckland New Zealand; ^3^ Pediatric Allergy and Pulmonology Units ‘Virgen de la Arrixaca’ University Children's Hospital University of Murcia and IMIB Bioresearch Institute Murcia Spain; ^4^ Population Health Research Institute St George's University of London London UK; ^5^ Centre for Global NCDs London School of Hygiene and Tropical Medicine London UK

**Keywords:** asthma, environment and hygiene hypothesis, epidemiology

## Abstract

**Background:**

Phase Three of the International Study of Asthma and Allergies in Childhood (ISAAC) measured the global prevalence of symptoms of asthma in children. We undertook comprehensive analyses addressing risk factors for asthma symptoms in combination, at both the individual and the school level, to explore the potential role of reverse causation due to selective avoidance or confounding by indication.

**Objective:**

To explore the role of reverse causation in risk factors of asthma symptoms.

**Methods:**

We compared two sets of multilevel logistic regression analyses, using (a) individual level exposure data and (b) school level average exposure (ie prevalence), in two different age groups. In individual level analyses, reverse causation is a possible concern if individual level exposure statuses were changed as a result of asthma symptoms or diagnosis. School level analyses may suffer from ecologic confounding, but reverse causation is less of a concern because individual changes in exposure status as a result of asthma symptoms would only have a small effect on overall school exposure levels.

**Results:**

There were 131 924 children aged 6‐7 years (2428 schools, 25 countries) with complete exposure, outcome and confounder data. The strongest associations in individual level analyses (fully adjusted) were for current paracetamol use (odds ratio = 2.06; 95% confidence interval 1.97‐2.16), early life antibiotic use (1.65; 1.58‐1.73) and open fire cooking (1.44; 1.26‐1.65). In school level analyses, these risk factors again showed increased risks.

There were 238 586 adolescents aged 13‐14 years (2072 schools, 42 countries) with complete exposure, outcome and confounder data. The strongest associations in individual level analyses (fully adjusted) were for current paracetamol use (1.80; 1.75‐1.86), cooking on an open fire (1.32; 1.22‐1.43) and maternal tobacco use (1.23; 1.18‐1.27). In school level analyses, these risk factors again showed increased risks.

**Conclusions & clinical relevance:**

These analyses strengthen the potentially causal interpretation of previously reported individual level findings, by providing evidence against reverse causation.

## 1 INTRODUCTION

Asthma is becoming increasingly important as a childhood disease on a global basis.^1^ The Global Asthma Report 2018 estimated that as many as 339 million people have asthma and that the burden of disability is high.^2^

The International Study of Asthma and Allergies in Childhood (ISAAC), using a simple and inexpensive standardized methodology,^3^, ^4^, ^5^ has documented a wide variation of asthma prevalence in different parts of the world,^6^, ^7^ and a number of papers have been published addressing the findings for individual risk factors, with several associations observed (see “Variables” below).^8^, ^9^, ^10^, ^11^, ^12^, ^13^, ^14^, ^15^, ^16^, ^17^, ^18^, ^19^, ^20^ However, these risk factors have not previously been considered together within the same analysis, so it is possible that some of the observed associations may be at least partially due to confounding by other risk factors.

The current paper represents the first comprehensive analyses to address these risk factors together, in order to fill this gap in the current knowledge. We have done this in two ways. Firstly, we have conducted a “standard” analysis using the individual level exposure data for each risk factor (eg maternal smoking). However, for some risk factors the cross‐sectional nature of the study means that such analyses may be subject to “reverse causation” if individual level exposure statuses were changed as a result of asthma symptoms or diagnosis. This may occur due to selective avoidance (eg if the child's mother stops smoking because the child has developed asthma) or “confounding by indication” (eg if exposures such as paracetamol or antibiotics are taken in response to symptoms which are related to the subsequent development of asthma).

As schools were the level of sampling in ISAAC, we have therefore conducted a second set of analyses using the school level average reported exposure (ie the prevalence; rather than the reported individual exposure) to each risk factor to attempt to avoid or minimize such biases. School level analyses may suffer from ecologic (community‐level) confounding, but reverse causation is perhaps less of a concern because individual changes in exposure status as a result of asthma symptoms would only have a small effect on overall school exposure levels. It is therefore of considerable interest to compare the individual level and school level analyses.

If reverse causation due to confounding by indication was exerting a major influence on the individual level associations, we would expect the associations to be much reduced at the school level. Conversely, if there was reverse causation due to selective avoidance, we would expect a stronger association at the school level, although this could also be due to contextual factors operating at the school level. Consistency of findings at the two levels thus provides indirect evidence against reverse causation and against strong contextual factors.

Biases may differ in different parts of the world, for example breastfeeding is more strongly associated with socio‐economic status in high‐income countries than in low‐ and middle‐income countries,^21^ hence there is a greater potential for confounding by socio‐economic status in the former. Therefore we additionally conducted analyses stratified by country‐level affluence to examine the extent to which associations and biases differed.

## 2 METHODS

### 2.1 Study

ISAAC Phase Three methods have been described in detail elsewhere^4^ and will be summarized briefly here. ISAAC Phase Three is a multi‐centre, multi‐country, cross‐sectional study of two age groups of schoolchildren (6‐7‐year‐old children and 13‐14‐year‐old adolescents) chosen from a random sample of schools in a defined geographical area.^3^, ^4^ The Phase Three survey took place in 2000‐2003 and included two standardized questionnaires. The first obtained data on symptoms of asthma, rhinoconjunctivitis and eczema and was identical to that used in Phase One of ISAAC.^6^, ^22^ The second, the environmental questionnaire, obtained data on a range of possible risk factors for the development of asthma and allergic disorders.^8^ The questionnaires can be found on the ISAAC website (http://isaac.auckland.ac.nz).

### 2.2 Variables

We considered the outcome of wheeze in the last 12 months, defined by a positive response to the question “Has your child/have you had wheezing or whistling in the chest in the past 12 months?” In many countries in the world, we find that most asthma (based on symptoms) has not been diagnosed, which is why ISAAC is based on symptoms. The ISAAC symptoms questionnaire validates well against doctor‐diagnosed asthma.^23^

The environmental questionnaires in the two age groups did not contain identical questions, so it was not possible to examine the same set of potential risk factors in each age group. In addition, we restricted our analyses to the risk factors which had shown associations with wheeze in the last 12 months in previous analyses at the individual level. For the younger age group, we included paracetamol use in the first year of life and in the past 12 months,^8^ antibiotic use in the first year of life,^20^ breastfeeding,^9^ cat in the home in the first year of life,^11^ regular contact with farm animals in the first year of life,^12^ truck traffic,^10^ fast food consumption,^13^ television viewing,^15^ parental smoking,^16^ cooking on an open fire^19^ and birth weight.^17^ For the older age group, we included truck traffic,^10^ fast food consumption,^13^ television viewing,^15^ parental smoking,^16^ paracetamol use in the past 12 months^24^ and open fire cooking.^19^

Most of the above risk factors were parameterised as binary variables from “yes/no” questions in the environmental questionnaire. The exceptions were as follows: paracetamol use in the past 12 months (at least once per month vs less than once per month), truck traffic (seldom or more frequently vs never), fast food consumption (once per week or more vs less than once per week), television viewing (at least 1 hour per day vs less than 1 hour per day) and birth weight (less than 2.5 kg vs at least 2.5 kg). Full definitions are in Table S1.

Sex was self‐reported as male/female, and the highest level of maternal education was recorded as primary, secondary, tertiary or missing/not stated.

Gross National Income (GNI) as of 2002 was obtained from the World Bank website^25^ where available, with gaps filled by the CIA World Factbook.^26^ Countries were classified as “affluent” or “non‐affluent” using a 2001 GNI value of US$9205 per capita as a cut‐off, which separates high‐income countries from low‐ and middle‐income countries.^27^

### 2.3 Statistical analyses

To be included in the analysis for a particular age group, centres had to include at least 1000 individuals and to have a response rate of >60% for children and >70% for adolescents. Analyses were conducted separately in the two age groups. Within each age group, schools with fewer than 10 individuals were excluded from the analysis.

All analyses were conducted using mixed effect (multilevel) logistic regression models. The four‐level hierarchical nature of the data (individuals [level 1], schools [level 2], centres [level 3] and countries [level 4]) was acknowledged by allowing random intercepts at levels 2, 3 and 4 in individual level models and by including random intercepts at levels 3 and 4 in school level models. Centres were self‐selected, whereas schools were randomly sampled within centres, making school the preferred level of analysis. Sex and maternal education were adjusted for as individual level confounders in all models.

Three different modelling approaches were used: (a) individual level, (b) school level and (c) hybrid fixed effects.^28^ However, results from the hybrid fixed effect models were very similar to those from the individual level and school level models, so they are not discussed further.

Individual level models related the individual level outcome to each individual level risk factor within schools. School level models related the individual level outcome to the school level average exposure (ie prevalence) of each risk factor. In these models, the estimated OR corresponding to the school level prevalence of the risk factor can be interpreted as the effect on the individual outcome of attending a school where all children are exposed compared to attending a school where no one is exposed.

Within each approach, models were fitted for: (a) each exposure of interest using the sub‐sample who had data present for wheeze, sex, maternal education and the given exposure (the “maximum sample”), (b) each exposure of interest using the sub‐sample who had data present for wheeze, sex, maternal education and all exposures of interest (the “common sample”) and (c) each exposure of interest mutually adjusted using the sub‐sample who had data present for wheeze, sex, maternal education and all exposures of interest (the “common sample”).

The extent of collinearity in the mutually adjusted models was examined by comparing the standard errors in the mutually adjusted model and the minimally adjusted model fitted to the same sub‐sample.^29^ There was no evidence of substantial collinearity.

Additionally, we ran the fully adjusted analyses separately for “affluent” and “non‐affluent” countries. We then separately tested for effect modification of each risk factor by country‐level affluence.

Analyses were conducted using Stata version 14.^30^

## 3 RESULTS

### 3.1 6‐7 year olds

**Figure 1:**
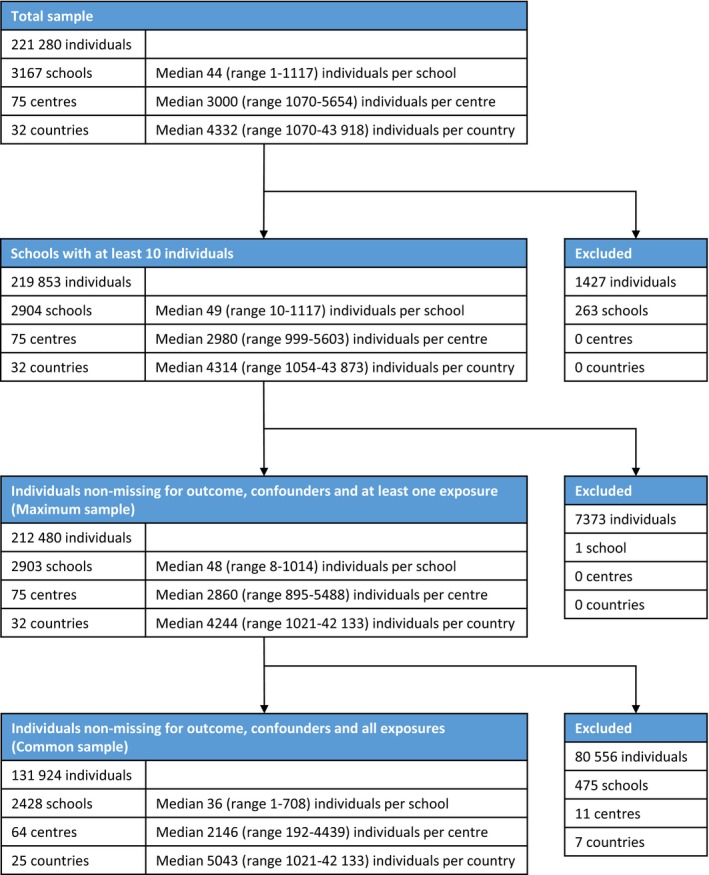
Data flowchart for 6‐7‐year‐old children

The 6‐7‐year‐old participants included 221 280 children from 75 centres which met the initial data quality criteria (at least 1000 children and a response rate of >60%). Of these, 212 480 children (from 2903 schools, 75 centres, 32 countries) were from schools with at least 10 children and had data present for wheeze, sex, maternal education and at least one of the exposures of interest so contributed to the analyses for one or more exposures (the “maximum sample”), with 131 924 children (from 2428 schools, 64 centres, 25 countries) having data present for all analysis variables (the “common sample”). See the data flowchart (Figure 1) for further details. Individual‐ and school level summary statistics are presented in Table S2 for the maximum sample and in Table 1 for the common sample.

**Table 1:** Summary statistics for variables and their prevalence in subjects who had data present for wheeze, sex, maternal education and all exposures of interest (the “common sample”)

Age group	Variable	Individual level (n = 131 924)	School level (n = 2428)	
		Prevalence (%)	Median prevalence (%)	Prevalence IQR (%)
6‐7 y	Wheeze in the last 12 mo	9.8	9.2	(4.7, 15.3)
	Low birthweight	8.1	6.1	(2.6, 10.7)
	Paracetamol (1st y)	65.4	70.6	(56.3, 83.9)
	Antibiotics (1st y)	56.2	57.6	(47.1, 66.0)
	Breastfed ever	81.3	85.2	(74.7, 93.7)
	Cat (1st y)	11.5	9.1	(3.8, 19.0)
	Farm animals (1st y)	10.2	9.1	(3.9, 16.7)
	Truck traffic (current)	79.2	84.5	(75.0, 91.3)
	Fast food (current)	39.9	31.6	(16.7, 50.0)
	Television (current)	79.8	84.4	(73.9, 91.6)
	Paternal tobacco (current)	32.3	34.4	(20.2, 48.3)
	Maternal tobacco (current)	15.3	14.2	(2.1, 30.7)
	Paracetamol (current)	18.3	14.7	(6.4, 28.0)
	Open fire cooking (current)	2.0	0.0	(0.0, 1.7)

Age group	Variable	Individual level (n = 238 586)	School level (n = 2072)	
		Prevalence (%)	Median prevalence (%)	Prevalence IQR (%)
13‐14 y	Wheeze in the last 12 mo	10.6	9.8	(5.0, 15.5)
	Truck traffic (current)	83.2	87.3	(79.5, 92.9)
	Fast food (current)	53.6	52.8	(38.9, 67.9)
	Television (current)	85.6	90.5	(81.7, 94.8)
	Paternal tobacco (current)	38.3	37.3	(23.5, 49.4)
	Maternal tobacco (current)	18.1	18.6	(3.4, 35.6)
	Paracetamol (current)	26.7	29.4	(17.3, 41.3)
	Open fire cooking (current)	5.2	0.7	(0.0, 3.0)

IQR, interquartile range.

Minimally adjusted associations in the common sample were broadly similar to those in the maximum sample (Tables 2 and S3). The strongest associations in the fully adjusted individual level analyses were for current paracetamol use (OR = 2.06, 95% CI 1.97‐2.16), antibiotic use in the first year of life (1.65; 1.58‐1.73) and open fire cooking (1.44; 1.26‐1.65) (Table 2).

**Table 2:** Effects of individual‐ and school level exposures on wheeze in the last 12 months for subjects who had data present for wheeze, sex, maternal education and all exposures of interest (the “common sample”). Mixed logistic regression models with random intercepts at the school, centre and country levels

Age group	Exposure	Individual level exposure		School level exposure	
		Minimally adjusted^a^ OR (95% CI)	Fully adjusted^b^ OR (95% CI)	Minimally adjusted^a^ OR (95% CI)	Fully adjusted^b^ OR (95% CI)
6‐7 y (n = 131 924)	Low birthweight	1.20 (1.12, 1.29)	1.12 (1.05, 1.21)	2.43 (1.60, 3.69)	2.13 (1.39, 3.25)
	Paracetamol (1st y)	1.75 (1.67, 1.84)	1.33 (1.27, 1.40)	1.42 (1.11, 1.82)	1.01 (0.78, 1.32)
	Antibiotics (1st y)	1.90 (1.83, 1.98)	1.65 (1.58, 1.73)	1.49 (1.17, 1.90)	1.38 (1.07, 1.78)
	Breastfed ever	0.91 (0.87, 0.96)	0.96 (0.91, 1.01)	0.80 (0.60, 1.09)	1.11 (0.82, 1.50)
	Cat (1st y)	1.29 (1.22, 1.37)	1.22 (1.15, 1.29)	1.44 (1.06, 1.94)	1.20 (0.88, 1.65)
	Farm animals (1st y)	1.24 (1.16, 1.31)	1.12 (1.06, 1.20)	1.47 (1.11, 1.94)	1.36 (1.00, 1.85)
	Truck traffic (current)	1.24 (1.17, 1.30)	1.17 (1.11, 1.23)	1.25 (0.97, 1.62)	1.04 (0.81, 1.33)
	Fast food (current)	1.14 (1.09, 1.19)	1.07 (1.03, 1.12)	1.80 (1.47, 2.20)	1.68 (1.37, 2.06)
	Television (current)	1.11 (1.06, 1.17)	1.04 (0.99, 1.10)	2.08 (1.61, 2.69)	1.80 (1.37, 2.37)
	Paternal tobacco (current)	1.20 (1.15, 1.25)	1.12 (1.07, 1.17)	1.51 (1.20, 1.89)	0.83 (0.63, 1.08)
	Maternal tobacco (current)	1.32 (1.25, 1.38)	1.20 (1.14, 1.27)	2.22 (1.72, 2.87)	1.83 (1.36, 2.47)
	Paracetamol (current)	2.35 (2.24, 2.46)	2.06 (1.97, 2.16)	2.05 (1.55, 2.71)	1.58 (1.18, 2.10)
	Open fire cooking (current)	1.44 (1.26, 1.65)	1.44 (1.26, 1.65)	1.95 (1.15, 3.29)	2.02 (1.16, 3.50)
13‐14 y (n = 238 586)	Truck traffic (current)	1.20 (1.15, 1.25)	1.16 (1.12, 1.21)	1.52 (1.09, 2.11)	1.28 (0.92, 1.79)
	Fast food (current)	1.11 (1.08, 1.15)	1.07 (1.04, 1.10)	1.36 (1.09, 1.71)	1.21 (0.96, 1.51)
	Television (current)	1.06 (1.01, 1.11)	1.02 (0.97, 1.07)	2.29 (1.56, 3.37)	2.01 (1.36, 2.96)
	Paternal tobacco (current)	1.19 (1.16, 1.23)	1.12 (1.08, 1.15)	0.85 (0.63, 1.13)	0.51 (0.37, 0.70)
	Maternal tobacco (current)	1.30 (1.26, 1.35)	1.23 (1.18, 1.27)	1.94 (1.39, 2.70)	2.51 (1.74, 3.61)
	Paracetamol (current)	1.83 (1.78, 1.89)	1.80 (1.75, 1.86)	2.43 (1.79, 3.29)	2.31 (1.71, 3.12)
	Open fire cooking (current)	1.31 (1.21, 1.41)	1.32 (1.22, 1.43)	0.98 (0.65, 1.48)	1.28 (0.85, 1.94)

a Adjusted for sex and mothers level of education.

b Additionally adjusted for all other variables in the table.

In the fully adjusted school level analyses, the associations for current paracetamol use (1.58; 1.18‐2.10), early life antibiotic use (1.38; 1.07‐1.78) and open fire cooking (2.02; 1.16‐3.50) were maintained (Table 2). Stronger associations were observed at the school level compared with the individual level for low birthweight (2.13; 1.39‐3.25 compared to 1.12; 1.05‐1.21), maternal tobacco use (1.83; 1.36‐2.47 compared to 1.20; 1.14‐1.27), fast food consumption (1.68; 1.37‐2.06 compared to 1.07; 1.03‐1.12) and early life farm animal exposure (1.36; 1.00‐1.85 compared to 1.12; 1.06‐1.20). An association was seen at the school level only for television viewing (1.80; 1.37‐2.37 compared to 1.04; 0.99‐1.10) (Table 2).

In the analyses stratified by country‐level affluence (Tables S4‐S5), there was strong evidence (*P* < 0.001) of effect modification at the individual level for early life exposure to cats (1.36; 1.26‐1.48 in non‐affluent countries vs 1.09; 1.00‐1.18 in affluent countries), early life exposure to farm animals (1.23; 1.14‐1.33 vs 0.96; 0.87‐1.06) and current paracetamol use (1.89; 1.79‐2.01 vs 2.38; 2.21‐2.56) (Table S4).

When using the school level prevalence (Table S5), there was again some evidence (*P* = 0.04) of effect modification of current paracetamol use (1.31; 0. 89‐1.92 in non‐affluent countries vs 2.32; 1.52‐3.55 in affluent countries). However, there was little evidence of a difference between affluent and non‐affluent countries for the associations of wheeze with cat and farm animal exposure in the first year of life. Several risk factors showed greater effect modification in the school level analysis than in the individual level analysis: maternal tobacco (3.30; 1.87‐5.83 in non‐affluent countries vs 1.49; 1.06‐2.10 in affluent countries in the school level analysis), antibiotics in the first year of life (1.13; 0.80‐1.61 vs 1.77; 1.22‐2.55) and paracetamol use in the first year of life (0.90; 0.63‐1.29 vs 1.30; 0.88‐1.93).

### 3.2 13‐14 year olds

**Figure 2:**
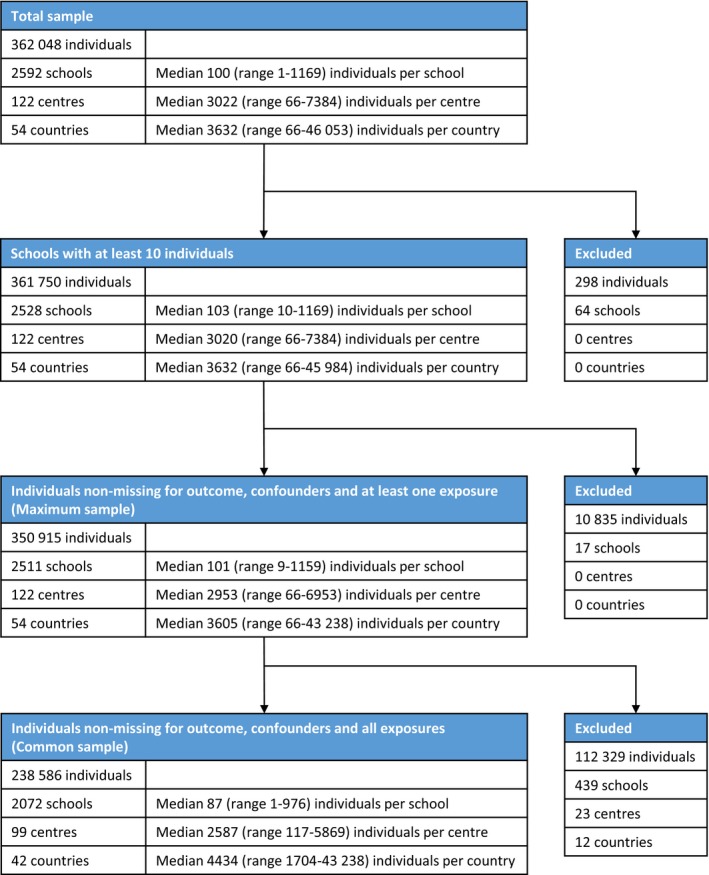
Data flowchart for 13‐14‐year‐old adolescents

The 13‐14‐year‐old participants included 362 048 adolescents from 122 centres which met the initial data quality criteria (at least 1000 adolescents and a response rate of >70%). Of these 350 915 adolescents (from 2511 schools, 122 centres, 54 countries) were from schools with at least 10 adolescents and had data present for wheeze, sex, maternal education and at least one of the exposures of interest so contributed to the analyses for one or more exposures (the “maximum sample”), with 238 586 adolescents (from 2072 schools, 99 centres, 42 countries) having data present for all analysis variables (the “common sample”). See the data flowchart (Figure 2) for further details. Individual‐ and school level summary statistics are presented in Table S2 for the maximum sample and in Table 1 for the common sample.

Minimally adjusted associations in the common sample were broadly similar to those in the maximum sample (Tables 2 and S3). The strongest associations in the fully adjusted individual level analyses were for current paracetamol use (1.80; 1.75‐1.86), cooking on an open fire (1.32; 1.22‐1.43) and maternal tobacco use (1.23; 1.18‐1.27) (Table 2).

In the fully adjusted school level analyses, the associations for current paracetamol use (2.31; 1.71‐3.12) and maternal tobacco use (2.51; 1. 74‐3.61) were maintained. Although the evidence for an association with cooking on an open fire was reduced, the point estimate was comparable to that in the individual level analysis (1.28; 0.85‐1.94) (Table 2). An association was also observed at the school level (but not the individual level) for television viewing (2.01; 1.36‐2.96). At the individual level, there was an association with paternal tobacco use (1.12; 1.08‐1.15), but this was in the other direction at the school level (0.51; 0.37‐0.70).

In the analyses stratified by country‐level affluence (Tables S4‐S5), there was evidence (*P* < 0.001) at the individual level that paracetamol use in the last 12 months was more strongly associated with wheeze in affluent countries (1.97; 1.85‐2.09) than non‐affluent (1.75; 1.69‐1.82) (Table S4). There was no evidence of effect modification at the school level (Table S5).

## 4 DISCUSSION

A number of papers have been published describing the association of asthma symptoms with individual level risk factors in ISAAC Phase Three.^8^, ^9^, ^10^, ^11^, ^12^, ^13^, ^14^, ^15^, ^16^, ^17^, ^18^, ^19^, ^20^ Here, we present the first comprehensive analyses to address these risk factors together in a multilevel framework and compare the individual level and school level findings to assess the possibility of various types of bias and confounding.

The associations we present here at the individual level (Table 2) generally confirm the results for recent wheeze in published ISAAC papers. However, the ORs do not correspond exactly with previous publications due to the following differences in analytical approach. Firstly, the ISAAC survey methodology involved cluster sampling (sampling schools, then selecting all children of the appropriate age within each selected school). In previous publications, no adjustment was made for within‐school clustering of risk factors. In our multilevel models, inclusion of school as a random intercept adjusts more formally for intra‐class correlation of both symptoms and exposures. This is a strength of the multilevel modelling approach.

Secondly, previous ISAAC Phase Three publications have adjusted for sex but not for socio‐economic status at the individual level, whereas we included individual level maternal education as a socio‐economic indicator in all models. Although maternal education is problematic to interpret as a socio‐economic indicator across diverse study centres from different countries and cultures, it is more likely to be valid for adjustment of socio‐economic confounding within local communities, such as school catchment areas, which is how it is used in our multilevel analyses.

Thirdly, previous ISAAC publications have adjusted for selected confounders (with a different set for each analysis), whereas we took a more comprehensive and harmonized approach in constructing our fully adjusted model. Comparison between the minimally adjusted and fully adjusted results in Table 2 confirms that the associations of wheeze with each risk factor are mutually independent, although in general there is some attenuation of the effects when all covariates are included. Some factors (eg paracetamol use in the first year of life) reduced markedly after confounder adjustment, indicating the possibility of residual confounding due to unmeasured confounders. Breastfeeding (in the younger children) and television viewing (in each age group) were the only individual level risk factors which became non‐significant after mutual adjustment, though the estimated associations in the minimally adjusted models were limited in magnitude prior to further adjustment.

A potential drawback of including multiple variables in a single model is a reduced sample size due to missing covariate data. About one‐third of the 6‐7‐year‐olds and about one‐quarter of the 13‐14‐year‐olds were excluded from the fully adjusted model due to incomplete risk factor information. However, comparison of results from the maximum sample with those from the common sample shows that findings were generally very similar for the subset of respondents with complete covariate data, suggesting that valid conclusions can be drawn from the “common sample” dataset.

It should also be noted that, whilst early life exposures are less prone to reverse causality than current exposures, recall errors (which may be biased with respect to disease status) are perhaps more likely to have affected early childhood exposures in an interview conducted when the child was 6‐7 years old.

An innovative feature of this paper is the presentation of associations of school level prevalence of risk factors with individual level wheeze. This type of population‐level analysis is potentially vulnerable to the “ecological fallacy,”^31^, ^32^ but this concept has several components, of which only one (ecological or population‐level confounding) applies in our study. We avoid other forms of ecological fallacy because the population‐level exposure (school level prevalence of each risk factor) was derived by aggregating individual level data, so the exposure measure relates directly to the schools actually participating in the study (not, for instance, a city‐wide or national average) and to the children for whom questionnaire data were returned (not, for instance, children of a different age or social group in the same area). We regard these as strengths of the multilevel analytical approach.

The school level associations shown in Table 2 generally maintained their direction on mutual adjustment, but the magnitude of the ORs (comparing the minimally adjusted and fully adjusted results) were less stable than the corresponding individual level associations (also in Table 2). Nevertheless, in the younger age group, significant school level associations were observed in the fully adjusted model with low birthweight, antibiotics in infancy, farm animal exposure in the first year, frequent fast food and television exposure, maternal smoking (but not paternal smoking) and current paracetamol use (but not paracetamol use in first year of life). In the older age group, significant school level associations were also observed with television viewing, maternal smoking and current paracetamol use.

The observed consistency of findings at the two levels provides indirect evidence against reverse causation and against strong contextual factors. Furthermore, since the spectrum of unmeasured confounders is likely to be different at the individual and population levels, consistency of results between the two levels provides additional reassurance against unmeasured confounding. Therefore, on both counts, cross‐level consistency strengthens the evidence for a causal relationship at the individual level.

Such cross‐level comparisons (Table 2) show a close similarity in ORs at the individual level and school level for current paracetamol exposure and wheeze in each age group. This is of particular interest as a causal interpretation of this association has been disputed, due to the possibility of reverse causation (due to confounding by indication for paracetamol use and wheezing in infancy, or due to aspirin avoidance by older children with asthma or their families).

ISAAC Phase Three findings for paracetamol in the first year of life have also been debated.^33^ At the individual level in the present study, we found an OR of 1.75 for paracetamol use in the first year of life, which reduced to 1.33 after adjusting for other risk factors; this is similar to the findings from the original report,^8^ which had ORs of 1.77 and 1.46 respectively. It has been suggested that this finding may be due to either residual confounding (given that more than one‐half of the excess risk has disappeared after adjustment for known confounders), or due to confounding by indication.^33^ This viewpoint is perhaps supported by the findings from our school level analyses, where the minimally adjusted association with paracetamol use in the first year of life (OR = 1.42) disappears on adjustment for other risk factors (OR = 1.01).

Another risk factor which might be prone to reverse causation (due to pet avoidance in allergic families) is cat exposure in infancy. Here, the school level association is somewhat stronger than the individual level association in the minimally adjusted models, as would be predicted from avoidance bias. However, after full adjustment the estimated associations are very similar.

In the older age group, we found associations with paternal tobacco smoking which differed in direction between the individual‐ and school level analyses. This was a surprising finding which we have been unable to satisfactorily explain.

Finally, stratified analyses identified some risk factors whose effects seemed to differ by country‐level affluence (Tables S4‐S5). In the younger age group, current paracetamol use was consistently (ie in both individual‐ and school level analyses) found to be a stronger risk factor for wheeze in affluent countries relative to non‐affluent countries. Cat and farm animal exposure in the first year of life were found to be stronger risk factors in non‐affluent countries (where there is perhaps less avoidance bias) in the individual level analysis. In the school level analysis, the affluence level‐specific associations similarly differed, though there was not statistical evidence for effect modification. In the older age group, current paracetamol use was again found to be a stronger risk factor for wheeze in affluent countries relative to non‐affluent countries, though only in the individual level analysis.

In conclusion, these multilevel analyses generally confirm previously reported child‐level findings for wheeze in ISAAC but, importantly, they provide additional evidence in favour of direct (rather than reverse) causation. This is the first comprehensive analysis of school level associations, which may be particularly relevant to public health policies, which aim to prevent asthma symptoms by modifying environment, lifestyle or medication use among whole communities, rather than individual children or their families.

## CONFLICT OF INTEREST

All authors declare no conflicts of interest.

## APPENDIX 1 ISAAC PHASE THREE STUDY GROUP

### 1.1

***ISAAC Steering Committee***: N Aït‐Khaled* (International Union Against Tuberculosis and Lung Diseases, Paris, France); HR Anderson (Population Health Research Institute, St George's, University of London, UK); MI Asher (Department of Paediatrics: Child and Youth Health, Faculty of Medical and Health Sciences, University of Auckland, New Zealand); R Beasley* (Medical Research Institute of New Zealand, Wellington, New Zealand); B Björkstén* (Institute of Environmental Medicine, Karolinska Institutet, Stockholm, Sweden); B Brunekreef (Institute of Risk Assessment Science, Universiteit Utrecht, Netherlands); J Crane (Wellington Asthma Research Group, Wellington School of Medicine, New Zealand); P Ellwood (Department of Paediatrics: Child and Youth Health, Faculty of Medical and Health Sciences, University of Auckland, New Zealand); C Flohr (Unit for Population‐Based Dermatology Research, St John's Institute of Dermatology, Guy's and St Thomas’ NHS Foundation Trust and King's College London, London, UK); S Foliaki* (Centre for Public Health Research, Massey University, Wellington, New Zealand); F Forastiere (Department of Epidemiology, Local Health Authority, Rome, Italy); L García‐Marcos (Pediatric Allergy and Pulmonology Units, Virgen de la Arrixaca University Children's Hospital, University of Murcia and Bio‐health Research Institute of Murcia (IMIB), Murcia, Spain); U Keil* (Institut für Epidemiologie und Sozialmedizin, Universität Münster, Germany); CKW Lai* (Department of Medicine and Therapeutics, The Chinese University of Hong Kong, SAR China); J Mallol* (Department of Respiratory Medicine, University of Santiago de Chile, Chile); EA Mitchell (Department of Paediatrics: Child and Youth Health, Faculty of Medical and Health Sciences, University of Auckland, New Zealand); S Montefort* (Department of Medicine, University of Malta, Malta), J Odhiambo†* (Centre Respiratory Diseases Research Unit, Kenya Medical Research Institute, Nairobi, Kenya); N Pearce (Faculty of Epidemiology and Population Health, London School of Hygiene and Tropical Medicine, London, UK); CF Robertson (Murdoch Children's Research Institute, Melbourne, Australia); AW Stewart (Population Health, Faculty of Medical and Health Sciences, University of Auckland, New Zealand); D Strachan (Population Health Research Institute, St George's, University of London, UK); E von Mutius (Dr von Haunerschen Kinderklinik de Universität München, Germany); SK Weiland† (Institute of Epidemiology, University of Ulm, Germany); G Weinmayr (Institute of Epidemiology and Medical Biometry, University of Ulm, Germany); HC Williams (Centre of Evidence‐Based Dermatology, University of Nottingham, UK); G Wong (Department of Paediatrics, Prince of Wales Hospital, Hong Kong, SAR China). *Regional Coordinators. †Deceased.

***ISAAC International Data Centre:*** MI Asher, TO Clayton†, P Ellwood, EA Mitchell, Department of Paediatrics: Child and Youth Health, and AW Stewart, School of Population Health, Faculty of Medical and Health Sciences, The University of Auckland, New Zealand. †Deceased.

***ISAAC Principal Investigators:*** **Argentina:** Dr CE Baena‐Cagnani*†, Catholic University of Córdoba (Córdoba), Dr M Gómez, Ayre Foundation; Hospital San Bernardo (Salta); **Barbados:** Dr ME Howitt*, Carlton Clinic (Barbados); **Belgium:** Prof J Weyler, University of Antwerp (Antwerp); **Bolivia:** Dra R Pinto‐Vargas*, Caja Petrolera de Salud (Santa Cruz); **Brasil:** Prof AJ da Cunha, Federal Universtity of Rio de Janeiro (Nova Iguaçu), Assoc Prof L de Freitas Souza, Universidade Federal da Bahia (Feira de Santana, Salvador, Vitória da Conquista); **Cameroon:** Prof C Kuaban*, University of Yaounde (Yaounde); **Canada:** Prof A Ferguson, University of British Columbia (Vancouver), Prof D Rennie, University of Saskatchewan (Saskatoon); **Channel Islands:** Dr P Standring, Princess Elizabeth Hospital (Guernsey); **Chile:** Dr P Aguilar, Hospital CRS El Pino (South Santiago), Dr L Amarales, Regional Hospital “Lautaro Navarro” (Punta Arenas), Dr LA Benavides, (Calama), Dra A Contreras, Hospital de Castro (Chiloe); **China:** Prof Y‐Z Chen*, Training Hospital for Peking University (Beijing, Tong Zhou), Assist Prof O Kunii, University of Tokyo (Tibet), Dr Q Li Pan, Xinjiang Children's Hospital (Wulumuqi), Prof NS Zhong, Guangzhou Institute of Respiratory Disease (Guangzhou); **Colombia:** Dr G Aristizábal, Instituto de Enfermedades Respiratorias del Niño S.A. (Bogotá), Dr AM Cepeda, Universidad Metropolitana (Barranquilla), Dr GA Ordoñez, Universidad Libre de Cali (Cali); **Ecuador:** Dr C Bustos, Hospital Alcivar (Guayaquil); **Estonia:** Dr M‐A Riikjärv*, Tallinn Children's Hospital (Tallinn); **Ethiopia:** Assoc Prof K Melaku, Addis Ababa University (Addis Ababa); **Fiji:** Dr R Sa'aga‐Banuve, UNICEF (Suva); **Finland:** Dr J Pekkanen*, National Public Health Institute (Kuopio County); **Gabon:** Dr IE Hypolite*, (Port‐Gentil); **Hungary:** Dr Z Novák, University of Szeged (Szeged), Dr G Zsigmond*, Senior Consultant (Svábhegy); **India:** Prof S Awasthi, King George's Medical University (Lucknow), Assoc Prof S Bhave, KEM Hospital Research Centre (Rasta Peth), Dr NM Hanumante, Ruby Hall Clinic (Pune), Dr KC Jain, Pioneer Medical Centre (Jodhpur), Dr MK Joshi, Panjat Hospital (Mumbai (16)), Dr VA Khatav, Dr Khatav's Mother and Child Hospital (Borivali), Dr SN Mantri, Jaslok Hospital & Research Centre (Mumbai (29)), Dr AV Pherwani, P.D. Hinduja Hospital and Medical Research Centre (Mumbai (18)), Prof S Rego, St John`s Medical College & Hospital (Bangalore), Prof M Sabir, Maharaja Agrasen Medical College Agroha (Bikaner), Dr S Salvi, Chest Research Foundation (Nagpur, Pimpri), Dr G Setty, (Chennai), Prof SK Sharma, All India Institute of Medical Sciences (New Delhi (7)), Prof V Singh, Asthma Bhawan (Jaipur), Dr T Sukumaran, PIMS Thiruvalla (Kottayam), Dr PS Suresh Babu, Bapuji Child Health Institute and Research Centre (Davangere); **Indonesia:** Prof Dr CB Kartasasmita, Padjajaran University (Bandung), Prof P Konthen†, Airlangga University (Bali), Dr W Suprihati, Diponegoro University (Semarang); **Iran:** Dr MR Masjedi*, National Research Institute of Tuberculosis and Lung Diseases (Rasht,Tehran); **Isle Of Man:** Dr A Steriu, Public Health Specialist, Information and Research (Isle of Man); **Ivory Coast:** Dr BN Koffi*, (Urban Cote d Ivoire); **Japan:** Dr H Odajima, National Hospital Organization Fukuoka Hospital (Fukuoka); **Kuwait:** Dr JA al‐Momen, Al‐Amiri Hospital (Kuwait); **Kyrgyzstan:** Dr C Imanalieva*, Kyrgyz Scientific Research Institute of Obstetrics and Pediatrics (Balykchi, Bishkek); **Lithuania:** Assoc Prof J Kudzyte*, Kaunas Medical University (Kaunas); **Malaysia:** Prof BS Quah, Melaka‐Manipal Medical College, (Kota Bharu), Dr KH Teh, Hospital Alor Setar (Alor Setar); **Malta:** Prof S Montefort*, University of Malta (Malta); **Mexico:** Dr M Baeza‐Bacab*, University Autónoma de Yucatán (Mérida), Dra M Barragán‐Meijueiro, CoMAAIPE (Ciudad de México (3)), Dra BE Del‐Río‐Navarro, Hospital Infantil de México (Ciudad de México (1)), Dr R García‐Almaráz, Hospital Infantil de Tamaulipas (Ciudad Victoria), Dr SN González‐Díaz, Hospital Universitario (Monterrey), Dr FJ Linares‐Zapién, Centro De Enfermedades Alergicas Y Asma de Toluca (Toluca), Dr JV Merida‐Palacio, Centro de Investigacion de Enfermedades Alergicas y Respiratorias (Mexicali Valley), Dra N Ramírez‐Chanona, COMPEDIA (Ciudad de México (4)), Dr S Romero‐Tapia, Hospital de Alta Especialidad del Niño (Villahermosa), Prof I Romieu, International Agency for Research on Cancer (Cuernavaca); **Morocco:** Prof Z Bouayad*, Service des Maladies Respiratoires (Boulmene, Casablanca, Marrakech); **New Zealand:** Prof MI Asher*, University of Auckland (Auckland), Dr R MacKay, Canterbury Health Laboratories (Nelson), Dr C Moyes, Whakatane Hospital (Bay of Plenty), Assoc Prof P Pattemore, University of Otago, Christchurch (Christchurch), Prof N Pearce, London School of Hygiene and Tropical Medicine (Wellington); **Nigeria:** Prof BO Onadeko, (Ibadan); **Panamá:** Dr G Cukier*, Hospital Materno Infantil Jose Domingo de Obaldia (David‐Panamá); **Peru:** Dr P Chiarella*, Universidad Peruana de Ciencias Aplicadas, UPC (Lima); **Philippines:** Prof F Cua‐Lim*†, University of Santo Tomas (Metro Manila); **Poland:** Assoc Prof A Brêborowicz, University of Medical Sciences (Poznan), Assoc Prof G Lis*, Jagiellonian University (Kraków); **Portugal:** Dra R Câmara, Centro Hospitilar do Funchal (Funchal), Dr ML Chiera, Hosp. Ped. Coimbra (Coimbra), Dr JM Lopes dos Santos, Hospital Pedro Hispano (Porto), Dr C Nunes, Center of Allergy and Immunology of Algarve (Portimao), Dr J Rosado Pinto*, Hospital da Luz (Lisbon); **Republic Of Macedonia:** Assoc Prof E Vlaski*, University Children's Clinic (Skopje); **Samoa:** Ms P Fuimaono V Pisi, (Apia); **SAR China:** Prof G Wong, Prince of Wales Hospital (Hong Kong 13‐14); **Singapore:** Assoc Prof DY Goh, National University of Singapore (Singapore); **South Africa:** Prof HJ Zar*, University of Cape Town (Cape Town); **South Korea:** Prof HB Lee*, Hanyang University College of Medicine (Provincial Korea, Seoul); **Spain:** Prof A Blanco‐Quirós, Facultad de Medicina (Valladolid), Dr RM Busquets, Universidad Autonoma de Barcelona (Barcelona), Dr I Carvajal‐Urueña, Centro de Salud de La Ería (Asturias), Dr G García‐Hernández, Hospital Universitario 12 de Octubre (Madrid), Prof L García‐Marcos*, University of Murcia and IMIB‐Arrxaca Research Institute (Cartagena), Dr C González Díaz, Universidad del País Vasco UPV /EHU (Bilbao), Dr A López‐Silvarrey Varela, Fundacion Maria Jose Jove (A Coruña), Prof M Morales‐Suárez‐Varela, Valencia University‐CIBERESP (Valencia), Prof EG Pérez‐Yarza, Universidad del Pais Vasco UPV/EHU (San Sebastián); **Sudan:** Prof OA Musa, National Ribat University (Khartoum); **Sultanate Of Oman:** Prof O Al‐Rawas*, Sultan Qaboos University (Al‐Khodh); **Syria:** Dr S Mohammad*, Tishreen University (Tartous), Prof Y Mohammad, National Center for Research and Training in Chronic Respiratory Diseases ‐ Tishreen University (Lattakia), Dr K Tabbah, Aleppo University Hospital (Aleppo); **Taiwan:** Dr JL Huang*, Chang Gung University (Taipei), Dr CC Kao, Kao‐Chun‐Chieh Clinic (Taoyuan); **Thailand:** Assoc Prof M Trakultivakorn, Chiang Mai University (Chiang Mai), Dr P Vichyanond*, Mahidol University (Bangkok); **Tokelau:** Dr T Iosefa*, Ministry of Health (Tokelau); **United Kingdom:** Dr M Burr†, Cardiff University Neuadd Meirionnydd (Wales), Prof D Strachan, Population Health Research Institute, St George's, University of London (Surrey/Sussex); **Uruguay:** Dra D Holgado*, Hospital Pereira Rossell (Montevideo), Dra MC Lapides, Hospital Paysandú (Paysandú); **USA:** Dr HH Windom, Asthma and Allergy Research Center (Sarasota); **Venezuela:** Dr O Aldrey*, Jefe del Instituto (Caracas). *National Coordinators. †Deceased.

***ISAAC National Coordinators not identified above:*** **Brazil:** Prof D Solé, Universidade Federal de São Paulo; **Canada:** Prof M Sears, McMaster University; **Chile:** Dra V Aguirre, Hospital CRS El Pino; **Ecuador:** Dr S Barba, AXXIS‐Medical Centre SEAICA; **India:** Dr J Shah, Jaslok Hospital & Research Centre; **Indonesia:** Prof Dr K Baratawidjaja, University of Indonesia; **Japan:** Prof S Nishima, The National Minami‐Fukuoka Chest Hospital; **Malaysia:** Assoc Prof J de Bruyne, University of Malaya; **Samoa:** Dr N Tuuau‐Potoi, Ministry of Health, Samoa; **SAR China:** Dr CK Lai, The Chinese University of Hong Kong; **Singapore:** Prof BW Lee, National University of Singapore; **Sudan:** Dr A El Sony, Epidemiological Laboratory (Epi‐Lab) for Public Health, Research and Development; **United Kingdom, Isle of Man:** Prof R Anderson, Population Health Research Institute, St George's, University of London.
