# Supplementary material for: Are environmental risk factors for current wheeze in the International Study of Asthma and Allergies in Childhood (ISAAC) phase three due to reverse causation?
Source: Clin Exp Allergy. 2019 Jan 23;49(4):430–41. doi: 10.1111/cea.13325 (PMC6487816; doi:10.1111/cea.13325)
Supplement: Supplementary file 1 [file CEA-49-430-s001.docx]

**SUPPORTING MATERIAL**

**Table S1: Risk Factor definitions**

| **Risk Factors for ages 6-7** | **Question (asked to parent)** | **Positive Response** |
| --- | --- | --- |
| Low birthweight | What was the weight of your child when he / she was born? | Less than 2.5kg |
| Paracetamol (1st year) | In the first 12 months of your child’s life, did you usually give paracetamol for fever? | Yes |
| Antibiotics (1st year) | In the first 12 months of your child’s life, did your child have any antibiotics? | Yes |
| Breastfed ever | Was your child breastfed? | Yes |
| Cat (1st year) | Did you have a cat in your home during the first year of your child’s life? | Yes |
| Farm animals (1st year) | In your child’s first year of life, did he / she have regular (at least once a week) contact with farm animals (e.g. cattle, pigs, goats, sheep or poultry)? | Yes |
| Truck traffic (current) | How often do trucks pass through the street where you live, on weekdays? | Seldom or more frequent |
| Fast food (current) | In the past 12 months, how often, on average did your child eat fast food / burgers? | At least once a week |
| Television (current) | During a normal week, how many hours a day (24 hours) does your child watch television? | At least one hour per day |
| Paternal tobacco (current) | Does your child’s father (or male guardian) smoke cigarettes? | Yes |
| Maternal tobacco (current) | Does your child’s mother (or female guardian) smoke cigarettes? | Yes |
| Paracetamol (current) | In the past 12 months, how often, on average, have you given your child paracetamol? | At least once per month |
| Open fire cooking (current) | In your house, what fuels are usually used for cooking? Electricity, gas, open fires, other | Any that include open fires |
|  |  |  |
| **Risk Factors for ages 13-14** | **Question (asked to child)** | **Positive Response** |
| Truck traffic (current) | How often do trucks pass through the street where you live, on weekdays? | Seldom or more frequent |
| Fast food (current) | In the past 12 months, how often, on average did you eat fast food / burgers? | At least once a week |
| Television (current) | During a normal week, how many hours a day (24 hours) do you watch television? | At least one hour per day |
| Paternal tobacco (current) | Does your father (or male guardian) smoke cigarettes? | Yes |
| Maternal tobacco (current) | Does your mother (or female guardian) smoke cigarettes? | Yes |
| Paracetamol (current) | In the past 12 months, how often, on average, have you taken paracetamol? | At least once per month |
| Open fire cooking (current) | In your house, what fuels are usually used for cooking? Electricity, gas, open fires, other | Any that include open fires |

**Table S2: Summary statistics for variables and their prevalence in subjects who had data present for wheeze, sex, maternal education and the given exposure** (**the “maximum sample”).**

| Age group | Variable | Individual-level | |  | School-level | | |
| --- | --- | --- | --- | --- | --- | --- | --- |
|  |  | n | Prevalence (%) |  | n | Median prevalence (%) | Prevalence IQR (%) |
| 6-7 years | Wheeze in the last 12 months | 212,480 | 10.4 |  | 2903 | 10.2 | (5.7, 16.7) |
|  | Low birthweight | 177,104 | 8.6 |  | 2601 | 6.3 | (3.2, 10.5) |
|  | Paracetamol (1st year) | 188,961 | 65.2 |  | 2635 | 69.6 | (56.2, 82.1) |
|  | Antibiotics (1st year) | 187,633 | 54.2 |  | 2715 | 56.0 | (46.3, 64.6) |
|  | Breastfed ever | 200,012 | 80.1 |  | 2753 | 84.5 | (73.5, 92.9) |
|  | Cat (1st year) | 197,501 | 11.8 |  | 2753 | 10.3 | (4.7, 19.3) |
|  | Farm animals (1st year) | 189,212 | 11.3 |  | 2686 | 9.6 | (4.3, 17.7) |
|  | Truck traffic (current) | 191,713 | 79.9 |  | 2781 | 85.4 | (76.2, 91.7) |
|  | Fast food (current) | 188,841 | 41.1 |  | 2850 | 33.3 | (18.6, 51.3) |
|  | Television (current) | 204,310 | 77.8 |  | 2855 | 83.8 | (74.1, 90.9) |
|  | Paternal tobacco (current) | 203,914 | 31.5 |  | 2800 | 33.3 | (19.2, 46.7) |
|  | Maternal tobacco (current) | 207,143 | 13.9 |  | 2833 | 13.9 | (2.9, 30.0) |
|  | Paracetamol (current) | 199,057 | 19.5 |  | 2786 | 16.4 | (7.6, 30.9) |
|  | Open fire cooking (current) | 192,631 | 3.1 |  | 2776 | 0.0 | (0.0, 2.3) |
| 13-14 years | Wheeze in the last 12 months | 350,915 | 11.4 |  | 2511 | 10.0 | (5.6, 15.8) |
|  | Truck traffic (current) | 318,661 | 82.5 |  | 2382 | 87.0 | (78.9, 92.5) |
|  | Fast food (current) | 320,128 | 55.2 |  | 2421 | 55.4 | (40.8, 69.5) |
|  | Television (current) | 339,823 | 85.3 |  | 2483 | 90.0 | (81.2, 94.3) |
|  | Paternal tobacco (current) | 310,700 | 37.5 |  | 2293 | 36.8 | (23.6, 48.3) |
|  | Maternal tobacco (current) | 338,740 | 17.9 |  | 2467 | 18.2 | (3.8, 33.3) |
|  | Paracetamol (current) | 323,051 | 28.8 |  | 2438 | 30.7 | (19.1, 43.7) |
|  | Open fire cooking (current) | 312,624 | 7.6 |  | 2355 | 1.2 | (0.0, 4.8) |

IQR = interquartile range.

**Table S3. Minimally adjusted^A^ effects of individual- and school-level exposures on wheeze in the last 12 months in subjects who had data present for wheeze, sex, maternal education and the given exposure (the “maximum sample”). Mixed logistic regression models with random intercepts at the school, centre and country levels.**

| Age group | Exposure | Individual-level exposure | |  | School-level exposure | |
| --- | --- | --- | --- | --- | --- | --- |
|  |  | n | OR (95% CI) |  | n | OR (95% CI) |
| 6-7 years | Low birthweight | 177,104 | 1.21 (1.14, 1.28) |  | 177,104 | 2.29 (1.56, 3.38) |
|  | Paracetamol (1st year) | 188,961 | 1.79 (1.72, 1.86) |  | 188,961 | 1.35 (1.07, 1.69) |
|  | Antibiotics (1st year) | 187,633 | 1.99 (1.92, 2.06) |  | 187,633 | 1.42 (1.14, 1.77) |
|  | Breastfed ever | 200,012 | 0.94 (0.91, 0.98) |  | 200,012 | 0.77 (0.59, 1.01) |
|  | Cat (1st year) | 197,501 | 1.37 (1.31, 1.44) |  | 197,501 | 1.84 (1.41, 2.40) |
|  | Farm animals (1st year) | 189,212 | 1.36 (1.30, 1.42) |  | 189,212 | 1.77 (1.38, 2.26) |
|  | Truck traffic (current) | 191,713 | 1.22 (1.17, 1.27) |  | 191,713 | 1.13 (0.90, 1.42) |
|  | Fast food (current) | 188,841 | 1.12 (1.08, 1.16) |  | 188,841 | 1.62 (1.36, 1.93) |
|  | Television (current) | 204,310 | 1.09 (1.05, 1.14) |  | 204,310 | 1.64 (1.32, 2.04) |
|  | Paternal tobacco (current) | 203,914 | 1.20 (1.16, 1.24) |  | 203,914 | 1.85 (1.50, 2.27) |
|  | Maternal tobacco (current) | 207,143 | 1.28 (1.23, 1.34) |  | 207,143 | 2.56 (2.03, 3.23) |
|  | Paracetamol (current) | 199,057 | 2.36 (2.28, 2.45) |  | 199,057 | 2.30 (1.81, 2.91) |
|  | Open fire cooking (current) | 192,631 | 1.30 (1.19, 1.43) |  | 192,631 | 3.28 (2.11, 5.07) |
| Age group | Exposure | Individual-level exposure | |  | School-level exposure | |
|  |  | n | OR (95% CI) |  | n | OR (95% CI) |
| 13-14 years | Truck traffic (current) | 318,661 | 1.22 (1.18, 1.26) |  | 318,661 | 1.64 (1.21, 2.21) |
|  | Fast food (current) | 320,128 | 1.12 (1.09, 1.14) |  | 320,128 | 1.37 (1.12, 1.69) |
|  | Television (current) | 339,823 | 1.05 (1.01, 1.08) |  | 339,823 | 2.09 (1.50, 2.91) |
|  | Paternal tobacco (current) | 310,700 | 1.20 (1.17, 1.23) |  | 310,700 | 0.81 (0.62, 1.05) |
|  | Maternal tobacco (current) | 338,740 | 1.33 (1.29, 1.37) |  | 338,740 | 1.78 (1.34, 2.38) |
|  | Paracetamol (current) | 323,051 | 1.84 (1.79, 1.89) |  | 323,051 | 2.25 (1.72, 2.94) |
|  | Open fire cooking (current) | 312,624 | 1.27 (1.19, 1.35) |  | 312,624 | 0.79 (0.58, 1.07) |

^A^Adjusted for sex and mothers level of education.

**Table S4. Fully adjusted^A^ effects of individual-level exposures on wheeze in the last 12 months in subjects who had data present for wheeze, sex, maternal education and all exposures of interest (the “common sample”), stratified by country-level affluence. Mixed logistic regression models with random intercepts at the school, centre and country levels.**

| Age group | Exposure | Affluent Countries  (n = 45,164) | |  | Non-Affluent countries  (n = 86,760) | | Effect  modification  p-value |
| --- | --- | --- | --- | --- | --- | --- | --- |
|  |  | Number exposed (%) | OR (95% CI) |  | Number exposed (%) | OR (95% CI) |  |
| 6-7 years | Low birthweight | 2,623 (5.8) | 1.17 (1.05, 1.31) |  | 8,051 (9.3) | 1.09 (0.99, 1.19) | 0.37 |
|  | Paracetamol (1st year) | 28,106 (62.2) | 1.38 (1.28, 1.49) |  | 58,210 (67.1) | 1.30 (1.22, 1.39) | 0.03 |
|  | Antibiotics (1st year) | 23,923 (53.0) | 1.71 (1.60, 1.82) |  | 50,193 (57.9) | 1.60 (1.51, 1.70) | 0.04 |
|  | Breastfed ever | 30,903 (68.4) | 1.03 (0.97, 1.10) |  | 76,316 (88.0) | 0.88 (0.82, 0.95) | 0.006 |
|  | Cat (1st year) | 6,762 (15.0) | 1.09 (1.00, 1.18) |  | 8,423 (9.7) | 1.36 (1.26, 1.48) | <0.001 |
|  | Farm animals (1st year) | 3,710 (8.2) | 0.96 (0.87, 1.06) |  | 9,762 (11.3) | 1.23 (1.14, 1.33) | <0.001 |
|  | Truck traffic (current) | 36,889 (81.7) | 1.12 (1.04, 1.21) |  | 67,569 (77.9) | 1.20 (1.12, 1.29) | 0.22 |
|  | Fast food (current) | 13,924 (30.8) | 1.08 (1.02, 1.15) |  | 38,767 (44.7) | 1.06 (1.00, 1.12) | 0.51 |
|  | Television (current) | 35,694 (79.0) | 1.05 (0.98, 1.13) |  | 69,573 (80.2) | 1.04 (0.96, 1.12) | 0.89 |
|  | Paternal tobacco (current) | 18,031 (39.9) | 1.10 (1.04, 1.17) |  | 24,595 (28.3) | 1.13 (1.06, 1.19) | 0.49 |
|  | Maternal tobacco (current) | 12,410 (27.5) | 1.19 (1.12, 1.28) |  | 7,839 (9.0) | 1.23 (1.12, 1.34) | 0.48 |
|  | Paracetamol (current) | 5,051 (11.2) | 2.38 (2.21, 2.56) |  | 19,030 (21.9) | 1.89 (1.79, 2.01) | <0.001 |
|  | Open fire cooking (current) | 256 (0.6) | 1.59 (1.17, 2.18) |  | 2,326 (2.7) | 1.40 (1.20, 1.63) | 0.60 |
| Age group | Exposure | Affluent Countries  (n=50,637) | |  | Non-Affluent Countries  (n=187,949) | | Effect  modification  p-value |
|  |  | Number exposed (%) | OR (95% CI) |  | Number exposed (%) | OR (95% CI) |  |
| 13-14 years | Truck traffic (current) | 43,519 (85.9) | 1.14 (1.05, 1.24) |  | 154,914 (82.4) | 1.17 (1.12, 1.23) | 0.11 |
|  | Fast food (current) | 25,567 (50.5) | 1.03 (0.97, 1.08) |  | 102,199 (54.4) | 1.09 (1.05, 1.12) | 0.34 |
|  | Television (current) | 46,066 (91.0) | 1.04 (0.94, 1.14) |  | 158,202 (84.2) | 1.01 (0.96, 1.07) | 0.63 |
|  | Paternal tobacco (current) | 20,731 (40.9) | 1.09 (1.03, 1.16) |  | 70,752 (37.6) | 1.13 (1.09, 1.17) | 0.09 |
|  | Maternal tobacco (current) | 15,167 (30.0) | 1.29 (1.21, 1.37) |  | 28,090 (14.9) | 1.20 (1.14, 1.25) | 0.69 |
|  | Paracetamol (current) | 13,453 (26.6) | 1.97 (1.85, 2.09) |  | 50,368 (26.8) | 1.75 (1.69, 1.82) | <0.001 |
|  | Open fire cooking (current) | 535 (1.1) | 1.14 (0.89, 1.45) |  | 11,930 (6.3) | 1.35 (1.24, 1.47) | 0.86 |

^A^Adjusted for sex, mother's level of education and all other variables in the table.

**Table S5: Fully adjusted^A^ effects of school-level exposures on prevalence on wheeze in the last 12 month in** **subjects** **who had data present for wheeze, sex, maternal education and all exposures of interest (the “common sample”), stratified by country-level affluence. Mixed logistic regression models with random intercepts at the school, centre and country levels.**

| Age group | Exposure | Affluent countries  (n = 45,164) | |  | Non-affluent countries  (n = 86,760) | | Effect  modification  p-value |
| --- | --- | --- | --- | --- | --- | --- | --- |
|  |  | Median prevalence (%) | OR (95% CI) |  | Median prevalence (%) | OR (95% CI) |  |
| 6-7 years | Low birthweight | 5.2 | 1.51 (0.73, 3.14) |  | 7.0 | 2.01 (1.16, 3.47) | 0.37 |
|  | Paracetamol (1st year) | 75.0 | 1.30 (0.88, 1.93) |  | 68.7 | 0.90 (0.63, 1.29) | 0.03 |
|  | Antibiotics (1st year) | 57.1 | 1.77 (1.22, 2.55) |  | 57.8 | 1.13 (0.80, 1.61) | 0.02 |
|  | Breastfed ever | 74.1 | 1.31 (0.92, 1.86) |  | 90.8 | 0.89 (0.53, 1.51) | 0.14 |
|  | Cat (1st year) | 9.3 | 0.95 (0.65, 1.41) |  | 9.0 | 1.50 (0.90, 2.51) | 0.08 |
|  | Farm animals (1st year) | 7.7 | 1.18 (0.76, 1.84) |  | 10.0 | 1.53 (0.99, 2.36) | 0.16 |
|  | Truck traffic (current) | 84.4 | 1.00 (0.68, 1.47) |  | 84.5 | 1.14 (0.82, 1.60) | 0.61 |
|  | Fast food (current) | 26.9 | 1.14 (0.80, 1.61) |  | 36.8 | 1.88 (1.45, 2.45) | 0.07 |
|  | Television (current) | 83.3 | 1.90 (1.32, 2.73) |  | 85.7 | 1.82 (1.19, 2.78) | 0.72 |
|  | Paternal tobacco (current) | 42.0 | 0.83 (0.59, 1.19) |  | 28.6 | 0.79 (0.54, 1.15) | 0.23 |
|  | Maternal tobacco (current) | 29.0 | 1.49 (1.06, 2.10) |  | 5.1 | 3.30 (1.87, 5.83) | 0.004 |
|  | Paracetamol (current) | 11.1 | 2.32 (1.52, 3.55) |  | 18.2 | 1.31 (0.89, 1.92) | 0.04 |
|  | Open fire cooking (current) | 0.0 | 0.82 (0.15, 4.51) |  | 0.0 | 2.15 (1.16, 3.97) | 0.20 |
| Age group | Exposure | Affluent countries  (n = 50,637) | |  | Non-affluent countries  (n = 187,949) | | Effect  modification  p-value |
|  |  | Median prevalence | OR (95% CI) |  | Median prevalence | OR (95% CI) |  |
| 13-14 years | Truck traffic (current) | 87.7 | 1.72 (0.69, 4.28) |  | 87.1 | 1.25 (0.86, 1.80) | 0.69 |
|  | Fast food (current) | 48.3 | 1.48 (0.91, 2.41) |  | 55.2 | 1.13 (0.87, 1.47) | 0.33 |
|  | Television (current) | 92.1 | 1.03 (0.41, 2.57) |  | 89.6 | 2.17 (1.40, 3.36) | 0.43 |
|  | Paternal tobacco (current) | 43.5 | 0.39 (0.21, 0.72) |  | 33.6 | 0.55 (0.38, 0.80) | 0.72 |
|  | Maternal tobacco (current) | 35.5 | 3.56 (1.86, 6.83) |  | 13.3 | 2.28 (1.47, 3.53) | 0.58 |
|  | Paracetamol (current) | 29.9 | 1.85 (1.07, 3.21) |  | 29.2 | 2.46 (1.73, 3.51) | 0.56 |
|  | Open fire cooking (current) | 0.0 | 1.23 (0.09, 16.44) |  | 0.8 | 1.31 (0.85, 2.04) | 0.84 |

^A^Adjusted for sex, mother's lev
